# Fibrinogen Increases Resveratrol Solubility and Prevents it from Oxidation

**DOI:** 10.3390/foods9060780

**Published:** 2020-06-12

**Authors:** Nikola Gligorijević, Mirjana Radomirović, Andreja Rajković, Olgica Nedić, Tanja Ćirković Veličković

**Affiliations:** 1 Institute for the Application of Nuclear Energy, Department for Metabolism, University of Belgrade, Banatska 31b, 11080 Belgrade, Serbia; nikolag@inep.co.rs (N.G.); olgica@inep.co.rs (O.N.); 2 Center of Excellence for Molecular Food Sciences & Department of Biochemistry, Faculty of Chemistry, University of Belgrade, Studentski trg 12–16, 11000 Belgrade, Serbia; radomirovicmirjana@chem.bg.ac.rs; 3 Faculty of Bioscience Engineering, Ghent University, 9000 Ghent, Belgium; Andreja.Rajkovic@ugent.be; 4 Department of Food Safety and Quality Management, Faculty of Agriculture, University of Belgrade, Nemanjina 6, 11080 Zemun-Belgrade, Serbia; 5 Global Campus, Ghent University, Yeonsu-gu, Incheon 406-840, Korea; 6 Serbian Academy of Sciences and Arts, Knez Mihailova 35, 11000 Belgrade, Serbia

**Keywords:** resveratrol, fibrinogen, binding, antioxidant, solubility

## Abstract

The French paradox describes a lower incidence of cardiovascular problems despite a high intake of saturated fats. This phenomenon was associated with higher consumption of red wine, as it was later discovered that the presence of antioxidants, including resveratrol, have beneficial effects. We hypothesized that resveratrol may have a more direct role in protection from harmful oxidation, presumably through binding to important proteins of the blood coagulation process. Spectrofluorimetry demonstrated that resveratrol is capable of binding to fibrinogen, the main protein in the coagulation process, which is also important as a food additive. Various spectroscopic methods determined that binding does not cause fibrinogen unfolding or destabilization since protein melting temperature remains unchanged. A mutually protective effect against the free radical-induced oxidation of polyphenol and fibrinogen was found. The presence of fibrinogen caused only a negligible masking effect of the antioxidative abilities of resveratrol, measured by a reduction of hexacyanoferrate (III), while greatly increasing its solubility in an aqueous environment, thus increasing its potential bioavailability. Due to its interaction with fibrinogen, resveratrol may serve as an antioxidant at the site of injury. The antioxidative effect of resveratrol may also protect and thus keep the desired characteristics of fibrinogen during the application of this protein as a food additive.

## 1. Introduction

Resveratrol (3,5,4′-trihydroxystilbene) is a well-known molecule that belongs to the stilbene group of polyphenols. Common sources of resveratrol are grapes, wine, peanuts, berries, and soy [1]. The idea that resveratrol may have beneficial effects on the cardiovascular system arose from the famous French paradox, according to which French people have lower incidences of cardiovascular disease (CVD) even though they consume a diet high in saturated fat [2]. In 2015, there were an estimated 422.7 million prevalent cases of cardiovascular diseases in the world. In the European Union (EU), CVD represents the leading cause of mortality, and each year over 1.8 million deaths are associated to CVD. From this number, there were 800,000 deaths in men and 1 million in women. The death proportion due to CVD in the EU ranges from 23% in France to 60% in Bulgaria in men, and from 25% in Denmark to 70% in Bulgaria in women. Ischemic heart disease (IHD) and strokes are the most frequent CVDs. In the latest available report, France was reported to have the lowest age-standardized death rate for IHD for both sexes, with 77 deaths per 100,000 in males and 32 deaths per 100,000 in females. Since low- and middle-income countries have significantly higher IHD death rates compared to high-income countries, these statistics should be treated with caution [3]. It was postulated that high wine consumption in France offers protection against CVD. It was also reported that there is a general association between decreased CVD incidence and mortality, and moderate alcohol consumption. This effect is linked with insulin sensitivity and lipid metabolism, assuming the causal alcohol–CVD association [4]. In 2015, almost three-quarters of French men consumed wine, compared to 60 per cent of women, with per capita consumption at 7.09 L expressed as pure alcohol (with a decreasing trend towards 2019). Later, it has been specified that the presence of resveratrol in wine is partially responsible for its beneficial effects due to its potent antioxidative activity, among other actions [2]. Resveratrol is well tolerated by humans and some studies report that plasma concentrations of this molecule can reach up to 4 μM depending on the supplemental dosages. Different studies have measured different concentrations of resveratrol in blood and they were found not to be in correlation with the initial concentration of this molecule in the supplement formulation [5]. Different methods used for the determination of resveratrol concentration also contribute to different values. Another important consideration is that all of these papers published concentrations of resveratrol present in the blood, while its actual concentration in tissues has still not fully been explored [5]. To put the role of resveratrol into perspective, bioavailability and pharmacokinetic/pharmacodynamic modelling should also be taken into account. When consumed orally, approximately 75% of resveratrol is absorbed. It is thought that its absorption mainly occurs by transepithelial diffusion. A very important issue concerning resveratrol is its bioavailability. The bioavailability of resveratrol is characterized by its rapid elimination. While its absorption process is highly effective, free resveratrol is present at low concentration in an organism due to the first hepatic step (first pass effect). Due to rapid metabolism in intestine and liver, the bioavailability of resveratrol falls below 1%. Additionally, the absorption of resveratrol is significantly delayed in the presence of food [6].

Plasma consists of over 100 proteins, the major ones being albumin, α-globulins, β-globulins, γ-globulins and fibrinogen. Fibrinogen is a 340 kDa glycoprotein composed of three pairs of chains with an (AαBβγ)2 structure [7] that circulates in the bloodstream at concentrations of 2 to 4 mg/mL (5.9–11.8 μM) [8]. Fibrinogen functions in the process of hemostasis, whereby during blood vessel injury the aggregation of platelets occurs through the interaction of fibrinogen with IIb/IIIa integrin on the surface of platelets, linking adjacent platelets and coagulating the blood (primary hemostasis) [9]. During secondary hemostasis, fibrinogen is converted to fibrin by thrombin, which removes fibrinopeptides A and B, forming fibrin monomers that associate with one another, creating a fibrin network that serves to mechanically reinforce the blood clot [7]. Many factors influence fibrin formation and, consequently, its degradation, including the types of fibrinogen chain [10], the glycosylation of fibrin [11], and the interactions with other proteins [7] and small molecules [12].

Fibrinogen has also been applied as a food additive in combination with thrombin, and it belongs to the category of food stabilizers [13,14]. For this application, both fibrinogen and thrombin are isolated from healthy slaughtered animals, including porcine (Sus scrofa domesticus) and bovine (Bos taurus) species that are consumed by humans. The meat industry uses the combination of thrombin and fibrinogen for fresh meat reconstitution in order to obtain the desired size and form. In order to do this, fibrin is formed from fibrinogen in the presence of thrombin. Thrombin hydrolyses fibrinogen and creates fibrin monomers by proteolytic cleavage of fibrinopeptides A and B. The formed fibrin monomers then polymerize and form a gel. The proteolytic action of thrombin also activates Factor XIII, which covalently binds fibrin monomers. Following the series of steps, several cross-links are made including fibrin–fibrin, fibrin–fibronectin, and fibrin–collagen. Due to the formation of cross-links and the gelation of fibrin, strong bonds are made between meat pieces [15]. Toxicological tests are not required since both fibrinogen and thrombin are obtained from edible parts of animals. The production of thrombin/fibrinogen enzyme preparation and its application as a food additive for reconstituting food can be safely used as concluded by EFSA’s Scientific Panel on Food Additives, Flavorings, Processing Aids and Materials in Contact with Food [13]. It is noteworthy that in low-pH products, such as fermented sausages, fibrinogen may have deleterious effects on protein functionality [16].

An important modification that drastically influences fibrinogen function is its degree of oxidation [17]. Fibrinogen is the blood protein that is most susceptible to oxidation [18], and its oxidation has been liked to both thrombotic and bleeding complications in cardiovascular-related diseases [19]. It is therefore attractive to investigate the ability of fibrinogen to bind molecules with antioxidative potential and to study the effects of those interactions. It is known that bovine fibrinogen spontaneously binds resveratrol with a binding constant of 1.11 × 10^4^. However, this interaction leads to conformational changes in fibrinogen secondary structure [20]. Having the French paradox in mind, we hypothesized that one possible mechanism behind the beneficial effects of resveratrol lies in the possible interaction of resveratrol with human fibrinogen and the subsequent protection of fibrinogen from oxidation thanks to the potent antioxidative effect of resveratrol.

## 2. Materials and Methods

### 2.1. Materials

All chemicals used were of analytical grade. Human fibrinogen was purchased from Sigma (Darmstadt, Germany) and further purified by dissolution in 50 mM phosphate buffer (pH 7.3), followed by precipitation with a saturated ammonium sulfate solution (reaching a final concentration of 25%), centrifugation and redissolution in the same buffer. This buffered solution of fibrinogen was then used for all subsequent experiments. The purity of the obtained fibrinogen was determined using reducing SDS-PAGE on 10% gel. Resveratrol was purchased from Carl Roth (Karlsruhe, Germany). Stock solutions of resveratrol were prepared by dissolution in 100% ethanol; the final concentration of ethanol was <2% in all experiments unless otherwise stated.

### 2.2. Spectrofluorometric Determination of the Fibrinogen/Resveratrol Affinity Constant

Spectrofluorometric analysis for determination of the affinity constant between fibrinogen and resveratrol was performed using a FluoroMax^®^-4 spectrofluorometer (Horiba Scientific, Japan). Fibrinogen (40 nM) was titrated with increasing concentrations of resveratrol (0–30 μM) at 25 °C. The protein was excited at 280 nm (excitation wavelength for both Tyr and Trp residues) and emission spectra were recorded from 290 to 450 nm (5 nm slit widths). The obtained fluorescence spectra were corrected by subtracting the fluorescence originating from resveratrol itself for each tested concentration. The change in the fibrinogen fluorescence intensity maximum at 345 nm was used to calculate the affinity constant. This fluorescence intensity was corrected for the inner filter effect using the following equation:

F_C_ = F_0_ × 10^(Aem + Aex)/2^ (1)

where F_C_ is the corrected fluorescence, F_0_ is the measured fluorescence, and Aem and Aex are the absorbances at the wavelengths of the emission (345 nm) and excitation (280 nm) peaks, respectively.

For the determination of the quenching type, the Stern–Volmer (SV) quenching constant was calculated according to the relationship [21]:

(2) $$\frac{F0}{F}=1+k_{q}\tau_{0}\left[Q\right]=1+K_{\mathrm{SV}}[Q]$$

where F_0_ and F are protein fluorescence intensity at 345 nm without and with resveratrol, respectively; k_q_ is the quenching rate constant of the biomolecule; τ_0_ is the average lifetime of the biomolecule without quencher (10^−8^ s); [Q] is the total quencher (resveratrol) concentration; and KSV is the SV quenching constant. The slope of the SV plot represents the SV quenching constant.

The binding constant, K_a_, between fibrinogen and resveratrol, was calculated using the following equation:

(3) $$\log\frac{F_{0}-F}{F}=-\mathrm{nlog}\frac{1}{\left[L\right]-\left[P\right]\frac{F_{0}-F}{F_{0}}}+{\mathrm{nlogK}}_{a}$$

where F_0_ and F are the emission signals of fibrinogen in the absence and in the presence of the ligand (resveratrol), respectively, while [L] and [P] represent the total concentrations of the ligand (resveratrol) and the protein (fibrinogen), respectively.

In a separate experiment, resveratrol (10 μM) was excited at 320 nm and its emission spectra in the absence and the presence of fibrinogen were recorded from 350 to 500 nm. Due to the large amounts of fibrinogen that would be required, no titration of resveratrol with protein was performed, and only one concentration of protein (10 μM) was used to demonstrate the consequence of complex formation on resveratrol emission.

### 2.3. Determination of Temperature Stability of the Fibrinogen/Resveratrol Complex

The influence of resveratrol on fibrinogen thermal stability was analyzed using the same equipment as described in Section 2.2. The temperature dependence of fibrinogen fluorescence, both alone (20 nM) and in the presence of resveratrol (20 μM) was followed by increasing the temperature from 25 to 75 °C at a rate of 1 °C/min (the equilibration period was set to 1 min). The results are presented as a ratio of the fluorescence intensities at 353 and 339 nm for the protein alone and at 363 and 349 nm for the fibrinogen/resveratrol complex since the binding of resveratrol causes a bathochromic shift of the fibrinogen emission maximum. As fibrinogen denaturation is characterized by a bathochromic shift of its emission maximum as well, the applied method gave results that were fitted into sigmoidal functions, with the inflection point representing the melting temperature of fibrinogen.

### 2.4. Analysis of Fibrinogen/Resveratrol Complex Formation Using Circular Dichroism (CD) Spectroscopy

The influence of fibrinogen/resveratrol complex formation on protein structure was investigated by recording near-UV and far-UV Circular Dichroism (CD) spectra of fibrinogen alone (3 μM) and fibrinogen in the presence of several resveratrol concentrations (5, 10, 15, 20, and 30 μM). The spectra were recorded using a J-815 spectropolarimeter (Jasco, Japan) at room temperature with a scan speed of 50 nm/min. The spectra were recorded from 260 to 320 nm (1 cm cell path) in the near-UV and from 185 to 260 nm (0.01 cm cell path) in the far-UV. The obtained spectra of fibrinogen in the presence of resveratrol were corrected by subtracting the spectra of resveratrol obtained at each concentration used, while the spectra of fibrinogen alone were corrected by subtracting the spectra of the buffer.

### 2.5. UV-VIS Analysis of Fibrinogen/Resveratrol Complex Formation

UV-VIS analysis was performed using a NanoDrop 2000c spectrophotometer (Thermo Scientific, Waltham, MA, USA). Spectra of fibrinogen (3 μM) in the absence and presence of resveratrol (30 μM) were recorded from 250 to 400 nm. In order to compare the spectral characteristics that originate from the protein alone with that of fibrinogen in the presence of resveratrol, the spectra obtained only from resveratrol were subtracted from the spectra of fibrinogen in the presence of resveratrol. A similar data transformation was also performed to determine whether there is also a change in the resveratrol spectra in the presence of fibrinogen; protein spectra were subtracted from the spectra of resveratrol in the presence of fibrinogen.

### 2.6. Effect of Fibrinogen on Resveratrol Solubility

Whether the presence of fibrinogen influenced resveratrol solubility was analyzed following a similar published procedure [22]. The samples for analysis consisted of 2 mM resveratrol in 13% ethanol in the absence or presence of fibrinogen (9 μM). After 5 min at room temperature, the mixtures were centrifuged at 10,000× *g* for 5 min and the supernatants were diluted 20-fold with a phosphate buffer. The amount of resveratrol present in the diluted supernatant was determined from the UV-VIS absorbance spectra recorded from 250 to 400 nm using the molar extinction coefficient of resveratrol (33,400 M^−1^ cm^−1^) at 312 nm.

### 2.7. Free Radical-Mediated Oxidation of Fibrinogen and Resveratrol

Fibrinogen oxidation induced by 2,2′-azobis(2-amidinopropane) dihydrochloride (AAPH) was followed by measuring the intrinsic fluorescence decay of fibrinogen after excitation at 280 nm. An aqueous AAPH solution was added to fibrinogen alone (100 nM) or to fibrinogen in the presence of resveratrol (200 nM); the final concentration of AAPH was 2.5 mM. Immediately upon AAPH addition, samples were placed in the spectrofluorometer and the fluorescence decay of fibrinogen at 345 nm was measured (5 nm slit widths, 280 nm excitation). The obtained results were corrected by subtracting the fluorescence contribution of resveratrol alone. The protective effect (PE), expressed in arbitrary units (a.u.) of resveratrol on fibrinogen oxidation was quantified using the following equation:

(4) $$\mathrm{PE}\left(\%\right)\frac{{\mathrm{AUC}}_{\mathrm{Fng}+R}\;-{\mathrm{AUC}}_{\mathrm{Fng}}}{{\mathrm{AUC}}_{\mathrm{Fng}+R}\;}\;\times \;100\;\%$$

where AUC_Fng+R_ represents the area under the curve obtained from oxidation of the fibrinogen/resveratrol mixture, while AUC_Fng_ represents the area under the curve obtained from oxidation of free fibrinogen.

To measure the free radical-mediated oxidation of resveratrol, UV-VIS spectroscopy was used to monitor the decrease in the absorption maximum of resveratrol at 305 nm over time in the presence of AAPH at 37 °C. To the resveratrol alone (15 μM), or in the presence of fibrinogen (7.5 μM), AAPH was added for a final concentration of 10 mM. After AAPH addition, samples were immediately placed in the UV-VIS spectrophotometer and the absorbance was monitored. Since a linear relationship was observed, the results are expressed as a difference between the initial and final measured absorbance.

### 2.8. Total Reducing Power of Fibrinogen, Resveratrol, and Their Complex

The reducing power of fibrinogen (7.5 μM), resveratrol (15 μM), and fibrinogen/resveratrol complex (7.5 μM fibrinogen and 15 μM resveratrol) samples was determined according to a published method [23]. To 40 μL of the sample solution, 100 μL of 0.2 M phosphate buffer (pH 6.6) and 100 μL of 1% potassium hexacyanoferrate (III) were added, followed by incubation at 50 °C for 20 min. After incubation, 20% trichloroacetic acid was added and the reaction mixture was centrifuged at 10,000× *g* for 8 min. The supernatant (100 μL) was mixed with 100 μL of deionized water and 12 μL of 1% FeCl_3_. After equilibrating for 10 min at room temperature, the absorbance was measured at 670 nm. The masking effect (ME) of the protein towards resveratrol was calculated using the following equation:

(5) $$\mathrm{ME}\left(\%\right)=\frac{A_{R}-A_{\mathrm{Fng}+R}}{A_{R}}\;\times \;100\;\%$$

where A_R_ and A_Fng+R_ are the measured absorbances at 670 nm in the absence and presence of fibrinogen, respectively.

### 2.9. Statistical Analysis of Data

All presented data are given as mean results of at least three independent measurements. Unless otherwise stated, they are all shown as means ± SD. For the comparison of data between two groups of samples in our experiments, Student’s t-test was used. Where it was necessary to compare three groups of data, a one-way ANOVA test was used. The results were considered significant if *p* value was below 0.05.

## 3. Results and Discussion

### 3.1. Binding Analysis of Fibrinogen and Resveratrol

**Figure 1:**
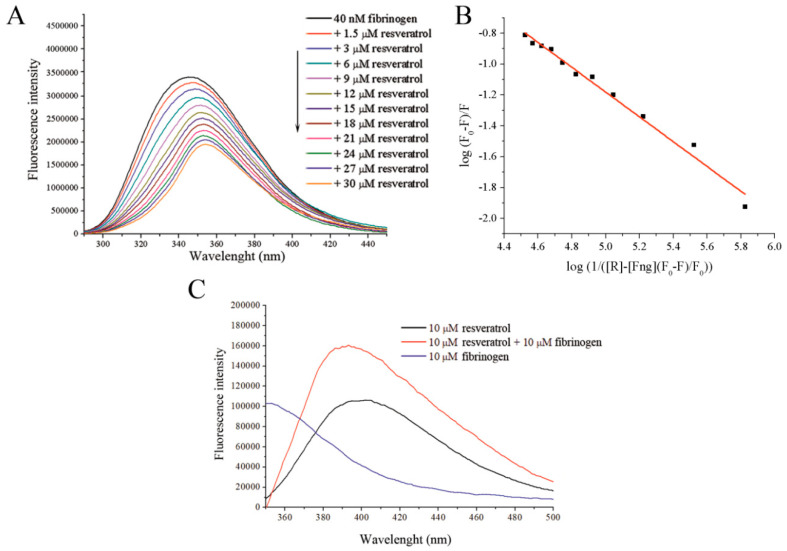
(**A**) Fluorescence emission quenching of fibrinogen upon its excitation at 280 nm in the presence of increasing resveratrol concentrations. (**B**) A Stern–Volmer plot obtained from fluorescence quenching data used for determination of the binding constant. (**C**) Resveratrol fluorescence emission after its excitation at 320 nm with and without fibrinogen.

Binding of resveratrol to fibrinogen quenches its intrinsic fluorescence in a dose-dependent manner (Figure 1A), within the tested range of concentrations. From the SV plot (Supplementary data Figure S1), and SV constant was calculated to be 4.4 × 10^3^ M^−1^ with bimolecular quenching rate constant of 4.4 × 10^11^ M^−1^, which is higher than the diffusion rates of biomolecules (10^10^ M^−1^ s^−1^) suggesting that a static type of quenching of fibrinogen occurs in the presence of resveratrol. Fluorescence quenching was used to determine the binding constant, Ka, which was calculated to be 2.58 ± 0.32 × 10^3^ M^−1^ (Figure 1B). A significant bathochromic shift (10 nm when 30 μM of resveratrol is present) is also observed in addition to a decrease in the fluorescence, suggesting that changes in the polarity of the fluorophore environment occur upon resveratrol binding. The occurrence of a bathochromic shift in the protein fluorescence suggests that there is a change in the environment of the Trp residues involved in the intrinsic fluorescence of fibrinogen, resulting in their exposure to a more polar environment [24]. Additional evidence supporting the occurrence of an interaction between resveratrol and fibrinogen is the nearly doubled intensity and significant hypsochromic shift (20 nm) of resveratrol fluorescence in the presence of fibrinogen compared to resveratrol alone when excited at 320 nm (Figure 1C).

**Figure 2:**
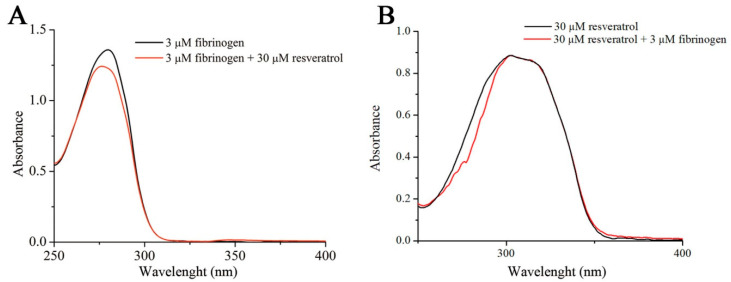
The effect of resveratrol on the UV-VIS spectra of fibrinogen (**A**). Overlapped UV-VIS spectra of resveratrol alone and resveratrol in the presence of fibrinogen (**B**).

Both the increase in the intrinsic resveratrol fluorescence and hypsochromic shift indicate that upon binding to fibrinogen, the environment polarity of resveratrol is reduced [25]. A similar effect was observed upon the interaction of resveratrol with β-lactoglobulin [22]. UV-VIS spectrophotometry reveals that fibrinogen absorbance at 280 nm decreases in the presence of resveratrol along with a small hypsochromic shift (Figure 2A). Therefore the polarity of Trp side residue in the fibrinogen molecule is increased upon interaction with resveratrol, which may suggest partial exposure of the fluorophore to the solvent due to unfolding of the protein. On the other hand, the presence of fibrinogen does not alter the resveratrol UV-VIS absorbance spectrum (Figure 2B), meaning that applied UV-VIS experimental conditions are not able to detect alterations of resveratrol environment as a consequence of its interaction with fibrinogen.

**Figure 3:**
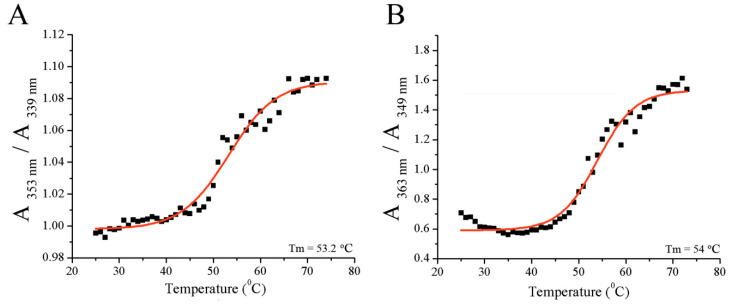
The determination of melting temperature of: (**A**) fibrinogen alone or (**B**) in the presence of resveratrol.

Similar melting temperatures were obtained for fibrinogen alone or in the presence of resveratrol (Figure 3A,B), suggesting that resveratrol does not significantly (*p* > 0.05) affect fibrinogen folding and stability. The melting temperature was 53.2 ± 0.6 °C for fibrinogen alone and 53.9 ± 0.48 °C for fibrinogen in the presence of resveratrol, which was similar to previously published results [8]. This melting temperature corresponds to the melting of the terminal D regions of fibrinogen [26].

### 3.2. Structural Analysis of Fibrinogen in Complex with Resveratrol

**Figure 4:**
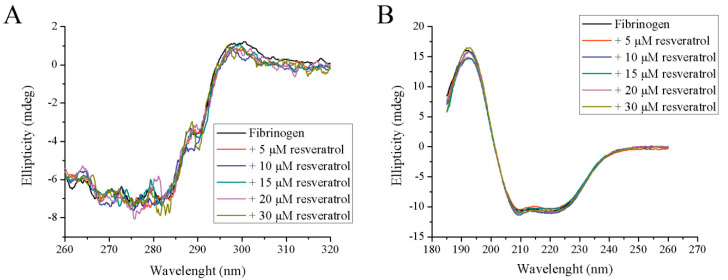
The effect of increasing concentrations of resveratrol on the tertiary and secondary structure of fibrinogen: (**A**) near-UV CD and (**B**) far-UV CD.

Although the results from spectrofluorometry and spectrophotometry may suggest some structural alteration in fibrinogen structure upon resveratrol binding, far-UV and near-UV CD spectroscopy failed to demonstrate any difference in both tertiary and secondary structures (Figure 4A,B). In the presence of all tested resveratrol concentrations, the far-UV and near-UV CD spectra are nearly the same. It seems that the amount of α-helixes, the most dominant secondary structure of fibrinogen, remains the same during resveratrol binding. Similar results were obtained when human fibrinogen was incubated with bilirubin [12]. However, the interaction of resveratrol with bovine fibrinogen decreases the α-helical content in the protein [20], suggesting that a different interaction mechanism occurs compared to human fibrinogen. The binding of resveratrol to some bovine milk proteins (e.g., β-lactoglobulin and β-casein) also did not have any significant effect on their secondary structure [27].

While it is already known that bovine fibrinogen is able to bind resveratrol [20], our results investigated this interaction using fibrinogen of human origin. When compared, the results point to some interesting differences. The affinity constant for human fibrinogen is lower almost by an order of magnitude. No structural differences are observed for human fibrinogen in the presence of resveratrol, which is not the case with bovine fibrinogen, where a reduction in the α-helical content of this protein was observed. Although fibrinogens from human and bovine origin are homologous, it seems that they are different enough to react differently with resveratrol.

### 3.3. Anti-Oxidative Effects of Fibrinogen/Resveratrol Complex

**Figure 5:**
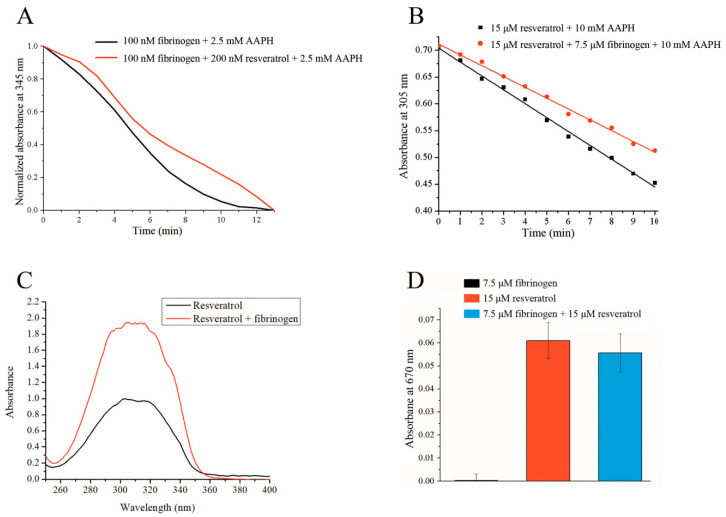
(**A**) The protective effect of resveratrol towards fibrinogen from 2,2′-azobis(2-amidinopropane) dihydrochloride (AAPH)-induced free radical oxidation measured by the fluorescence decay of fibrinogen. (**B**) The protective effect of fibrinogen towards resveratrol from AAPH-induced free radical oxidation measured by the reduction of resveratrol absorbance at 305 nm. (**C**) The effect of fibrinogen on resveratrol solubility in an aqueous environment. (**D**) The reducing ability (absorbance) of free fibrinogen, free resveratrol, and fibrinogen/resveratrol complex.

Under the experimental conditions tested, protection from AAPH-induced free radical oxidation is mutual, whereby resveratrol is able to protect fibrinogen from oxidation, and vice versa. To measure the protective effect of resveratrol on fibrinogen oxidation, we determined the area under the curves obtained from the decrease in intrinsic fibrinogen fluorescence in the presence of AAPH. The measured area under the curve (expressed in a.u.) in the presence of resveratrol was 6.1 ± 0.5, which was significantly higher (*p* < 0.05) than the area under the curve for fibrinogen alone, which was 4.8 ± 1.8. In the presence of resveratrol, the area was on average 27% larger compared to fibrinogen alone (Figure 5A). It can clearly be seen from Figure 5A that in the presence of resveratrol, an initial short lag phase is observed that is missing when only fibrinogen is present. The oxidation of resveratrol displays a linear relationship over time (Figure 5B), allowing the results to be expressed as slopes. As can be seen from Figure 5B, the slope of the linear graph obtained in the presence of fibrinogen (k = −0.020 ± 0.000014) is not as steep as the slope for resveratrol alone (k = −0.026 ± 0.000292), indicating that fibrinogen is capable of protecting resveratrol from free radical-induced oxidation. The obtained slopes were significantly different (*p* < 0.05).

The results of this study suggest the possibility that the interaction of resveratrol with fibrinogen protects resveratrol from oxidation, allowing it to reach the site of injury in its antioxidative form. As a fibrin clot starts to assemble, resveratrol may either be initially released due to fibrin monomer association or released later as a consequence of fibrin degradation by plasmin. In either scenario, its antioxidative effect can be realized in close proximity to the injury, positively influencing the local healing process.

Another important consequence of this interaction is the protection of the fibrinogen protein itself. Extensive studies of fibrinogen oxidation indicate the detrimental effects of oxidation on its function, including its involvement in many pathologic conditions that are characterized as having a high risk for developing thrombotic complications. Thus far, both in vivo and in vitro studies have shown that the oxidation of fibrinogen alters its coagulation properties, such as through the formation of thinner fibrin fibers and the reduction of its porosity [28,29,30]. For instance, glyco-oxidation from diabetes affects fibrinogen in such a way that activation of plasmin, a key protease for its degradation, is impaired on the surface of fibrinogen [31]. As a consequence, all of these effects can alter fibrinogen and make it more thrombogenic.

It is therefore important to study the interactions and effects of active food-derived components that have antioxidative potential with fibrinogen. These components must protect fibrinogen from harmful oxidation while also being benign or beneficial to its hemostatic function. Resveratrol does not interfere with fibrinogen hemostatic properties, including both fibrin formation and fibrinolysis [32], making it likely that its consumption will only have a positive protective effect on this protein. Another important aspect of resveratrol is its inhibitory effect on platelet activation, a phenomenon that is present under inflammation conditions and oxidative stress [33].

Protecting fibrinogen through its interaction with bioactive food components may also be important in its application as a food additive. In addition to protective antioxidative effect, these interactions may also lead to the formation of fibrin gels with more desirable characteristics suitable for their usage as food stabilisers.

Elevated fibrinogen levels in plasma are considered as a risk factor for poor prognosis in many human diseases, and this protein is also designated as one of the damage-associated molecular pattern (DAMPs) proteins. The consequences of an upregulated fibrinogen are yet to be elucidated, but the impairment of mitochondria may be one of them. The amelioration of this effect is possible by resveratrol, which is known to improve cell-dependent mitochondrial functions under in vitro and in vivo conditions, including an increased mitochondrial bioenergetics. Moreover, an increased plasma fibrinogen level is a prothrombotic factor. In non-alcoholic fatty liver disease (NAFLD), an upregulation of a fibrinogen is an independent risk factor of CVD. Studies with trans-resveratrol have shown that this polyphenol reduces the liver fat and hepatic enzymes such as glutamate pyruvic transaminase (SGPT) and gamma-glutamyl transpeptidase (g-GT), and it also ameliorates insulin resistance, thus preventing and reducing liver damage in NAFLD [34,35,36,37].

### 3.4. Effect of Fibrinogen on Resveratrol Solubility

Another very important aspect of any medication or food supplement is its bioavailability in living systems. Because the solubility of resveratrol in aqueous environments such as blood is poor [38], it is necessary that some mechanisms are present to transport it in the bloodstream or increase its solubility while in circulation. For instance, cyclodextrins are able to form complexes with resveratrol and increase its solubility in aqueous solutions while some were able to increase its light stability [39].

The experimental conditions described in Section 2.6 resulted in the formation of precipitates from both reaction mixtures after equilibration for five minutes at room temperature, though more precipitate was observed in the mixture without fibrinogen. After the centrifugation and dilution of the supernatant, the UV-VIS spectra of the samples were recorded, whereby the absorbance of the mixture that contained fibrinogen was twice as intense as the one without protein (Figure 5C), suggesting that the presence of fibrinogen increases resveratrol solubility in an aqueous environment, thus increasing its bioavailability. Thye measured concentration of resveratrol was 0.57 ± 0.012 mM in the absence of fibrinogen and significantly higher (*p* < 0.05) in the presence of fibrinogen. It was calculated to be 1.13 ± 0.012 mM. A similar effect was observed for β-lactoglobulin [22].

### 3.5. Reducing Ability of Fibrinogen/Resveratrol Complex

Under the presented experimental conditions, fibrinogen itself is not capable of reducing hexacyanoferrate (III), demonstrating that all antioxidative capabilities can be attributed to resveratrol (Figure 5D). The masking effect is usually a normal consequence of protein-ligand interactions [23,40], and it is characterized by lowering the antioxidative ability of bioactive ligands. While this may initially seem like a negative consequence of the interaction, the degradation of a protein may release bound ligand with its full antioxidative potential. The presence of fibrinogen creates a very small masking effect on the reducing ability of resveratrol (8%). A statistical analysis indicated that this effect was not significant (*p* > 0.05) as determined by a one-way ANOVA test. This means that resveratrol has almost full antioxidative potential even when it is bound to fibrinogen. In addition, the initial interaction with fibrinogen protects resveratrol, thus prolonging its bioactivity. Upon fibrinogen degradation, a fully active resveratrol can be released.

## 4. Conclusions

Binding between fibrinogen and resveratrol was detected, and together they provide mutually beneficial effects towards one another. This agrees with our hypothesis as one possible mechanistic insight into the famous French paradox that is characterized by the relatively few occurrences of cardiovascular complications despite the consumption of a high-fat diet. Here, we have demonstrated at the molecular level that a polyphenol of red wine, resveratrol, may bind and protect fibrinogen from harmful oxidation. Fibrinogen also increases the solubility of resveratrol in aqueous environments and provides protection from oxidation, thus increasing the bioavailability of resveratrol and its antioxidant potential.

## Supplementary Materials

The following are available online at https://www.mdpi.com/2304-8158/9/6/780/s1, Figure S1: Stern-Volmer (SV) plot used to calculate SV constant and bimolecular quenching rate constant.
