# Supplementary material for: Fibrinogen Increases Resveratrol Solubility and Prevents it from Oxidation
Source: Foods. 2020 Jun 12;9(6):780. doi: 10.3390/foods9060780 (PMC7353596; doi:10.3390/foods9060780)
Supplement: Supplementary file 1 [file foods-09-00780-s001.pdf]

# Supplementary data

## Interactions and effects of the red wine polyphenol resveratrol binding to human fibrinogen

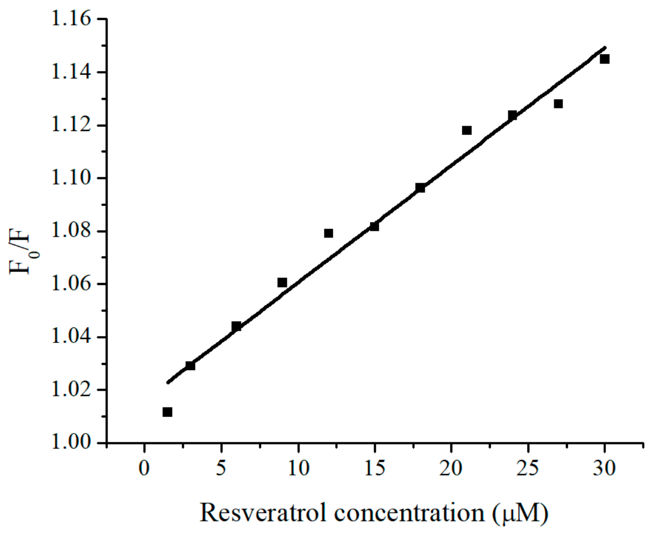


Figure S1. Stern-Volmer (SV) plot used to calculate SV constant and bimolecular quenching rate constant
